# Clinical long-time course, novel mutations and genotype-phenotype correlation in a cohort of 27 families with *POMT1*-related disorders

**DOI:** 10.1186/s13023-019-1119-0

**Published:** 2019-07-16

**Authors:** Tobias Geis, Tanja Rödl, Haluk Topaloğlu, Burcu Balci-Hayta, Sophie Hinreiner, Wolfgang Müller-Felber, Benedikt Schoser, Yasmin Mehraein, Angela Hübner, Birgit Zirn, Markus Hoopmann, Heiko Reutter, David Mowat, Gerhard Schuierer, Ulrike Schara, Ute Hehr, Heike Kölbel

**Affiliations:** 1 Department of Pediatric Neurology, Klinik St. Hedwig, University Children’s Hospital Regensburg (KUNO), Steinmetzstr. 1-3, 93049 Regensburg, Germany; 2 Center for Human Genetics, Regensburg, Germany; 3 Department of Pediatric Neurology, Faculty of Medicine, Hacettepe University, Ankara, Turkey; 4 Department of Medical Biology, Faculty of Medicine, Hacettepe University, Ankara, Turkey; 5 Department of Pediatric Neurology, University Hospital Munich, Munich, Germany; 6 Friedrich-Baur-Institut, Neurologische Klinik, Ludwig-Maximilians-Universität München, Munich, Germany; 7 Institute of Human Genetics, University Hospital, Ludwig-Maximilians-Universität München, Munich, Germany; 8 Pediatrics, University Hospital, Technical University Dresden, Dresden, Germany; 9 Genetic Counselling and Diagnostic, Genetikum Stuttgart, Stuttgart, Germany; 10 Department of Obstetrics and Gynaecology, University of Tuebingen, Tuebingen, Germany; 11 Department of Neonatology, University Hospital of Bonn, Bonn, Germany; 12 Department of Clinical Genetics, Sydney Children’s Hospital Randwick, Sydney, Australia; 13 Department of Neuroradiology, University of Regensburg, Regensburg, Germany; 14 Department of Pediatric Neurology, University Hospital Essen, Essen, Germany; 15 Department of Human Genetics, University of Regensburg, Regensburg, Germany

**Keywords:** POMT1, Dystroglycan, Walker-Warburg syndrome, Limb girdle muscular dystrophy, Muscle-eye-brain disease, Lissencephaly, Hydrocephalus, Occipital encephalocele

## Abstract

**Background:**

The protein O-mannosyltransferase 1, encoded by the *POMT1* gene, is a key enzyme in the glycosylation of α-dystroglycan. *POMT1*–related disorders belong to the group of dystroglycanopathies characterized by a proximally pronounced muscular dystrophy with structural or functional involvement of the brain and/or the eyes. The phenotypic spectrum ranges from the severe Walker-Warburg syndrome (WWS) to milder forms of limb girdle muscular dystrophy (LGMD). The phenotypic severity of *POMT1*-related dystroglycanopathies depends on the residual enzyme activity. A genotype-phenotype correlation can be assumed.

**Results:**

The clinical, neuroradiological, and genetic findings of 35 patients with biallelic *POMT1* mutations (15 WWS, 1 MEB (muscle-eye-brain disease), 19 LGMD) from 27 independent families are reported. The representative clinical course of an infant with WWS and the long-term course of a 32 years old patient with LGMD are described in more detail. Specific features of 15 patients with the homozygous founder mutation p.Ala200Pro are defined as a distinct and mildly affected LGMD subgroup. Ten previously reported and 8 novel *POMT1* mutations were identified. Type and location of each of the *POMT1* mutations are evaluated in detail and a list of all *POMT1* mutations reported by now is provided. Patients with two mutations leading to premature protein termination had a WWS phenotype, while the presence of at least one missense mutation was associated with milder phenotypes. In the patient with MEB-like phenotype two missense mutations were observed within the catalytic active domain of the enzyme.

**Conclusions:**

Our large cohort confirms the importance of type and location of each *POMT1* mutation for the individual clinical manifestation and thereby expands the knowledge on the genotype-phenotype correlation in *POMT1*-related dystroglycanopathies. This genotype-phenotype correlation is further supported by the observation of an intrafamiliar analogous clinical manifestation observed in all affected 13 siblings from 5 independent families. Our data confirm the progressive nature of the disease also in milder LGMD phenotypes, ultimately resulting in loss of ambulation at a variable age. Our data define two major clinical *POMT1* phenotypes, which should prompt genetic testing including the *POMT1* gene: patients with a severe WWS manifestation predominantly present with profound neonatal muscular hypotonia and a severe and progressive hydrocephalus with involvement of brainstem and/or cerebellum. The presence of an occipital encephalocele in a WWS patient might point to POMT1 as causative gene within the different genes associated with WWS. The milder LGMD phenotypes constantly show markedly elevated creatine kinase values in combination with microcephaly and cognitive impairment.

**Electronic supplementary material:**

The online version of this article (10.1186/s13023-019-1119-0) contains supplementary material, which is available to authorized users.

## Background

Muscular dystrophies with defective O-glycosylation of α-dystroglycan (dystroglycanopathies) are a genetically heterogeneous group of autosomal recessive inherited disorders with a broad clinical spectrum. Structural or functional involvement of the central nervous system (CNS) and/or the eyes is considered a characteristic feature of dystroglycanopathies [1]. Proper glycosylation of α-dystroglycan (aDG) is necessary for this glycoprotein to bind to extracellular matrix components like laminin, perlecan and agrin [2, 3]. The protein O-mannosyltransferase 1, encoded by the *POMT1* gene, is a glycosyltransferase catalyzing the transfer of the initial O-mannose residue to a Serine or Threonine residue and thus the first step in glycosylation of aDG. In 2002, mutations of the *POMT1* were found to be associated with Walker-Warburg syndrome (WWS) [4], considered the most severe subgroup of dystroglycanopathies. Characteristic brain malformations in WWS consist of supratentorial neuronal migration disorders (NMD) with cobblestone lissencephaly as a typical hallmark and infratentorial involvement with cerebellar hypoplasia and/or brainstem hypoplasia. The presence of cobblestone lissencephaly and cerebellar involvement as constant findings were described in 1989 as diagnostic criteria for WWS brain malformations [5] and later defined a distinct feature to distinguish WWS from muscle-eye-brain disease (MEB) [6]. From 2005 on, the phenotypic spectrum of *POMT1*-dependent diseases was expanded as milder forms of limb girdle muscular dystrophy (LGMD2K, 1; MDDGC1; OMIM 609308) and congenital muscular dystrophy (CMD type B1; MDDGB1; OMIM 613155) were described [7–10]. A distinct phenotype in Turkish patients with mild retardation without structural brain malformation could be linked to the ancestral founder mutation p.Ala200Pro [8]. The phenotypic severity of *POMT1*-related conditions depends on the genotype. While patients with 2 truncating mutations have a WWS phenotype [4, 11] the presence of at least one missense mutation is associated with a milder phenotype [7, 10, 12, 13].

In this study the clinical, neuroradiological, and molecular genetic findings of 35 *POMT1* patients from 27 independent families of various ethnic origins are described in detail with focus on the mutation type and location in order to improve genetic counseling for the affected families on disease course and long-term prognosis. Moreover, the characteristic clinical course of an infant with WWS and the long-term course of a patient with LGMD and remarkably late genetic diagnosis at the age of 32 years are described in more detail. Clinical long-term features of the subgroup of 15 patients with the homozygous founder mutation p.Ala200Pro are discussed.

## Results

### Clinical findings

Our cohort consisted of 35 patients from 27 unrelated families. Eight of these patients had been published previously [8, 11, 14, 15]. Sixteen families were of Turkish origin; there was 1 Indonesian, 1 gipsy and 1 African family and 8 families with German origin. The patient’s age at the time of diagnosis ranged from prenatal diagnosis in the fetuses to 32 years of age in a LGMD patient. Considering the proposed classifications of Godfrey et al. and Straub et al. we classified 15 patients as WWS (Walker-Warburg syndrome), 1 patient as MEB-like phenotype (muscle-eye-brain disease-like) and 19 patients as LGMD-MR phenotype (LGMD with mental retardation) [1, 16]. An overview of the clinical features of the patients is given in Table 1.

**Table 1:** Summary of the clinical and neuroradiological data

Pat	Diagnosis	Consanguinity	Age at onset	Ethnic origin	maximum CK	Muscle histology	Motor ability	Muscle contractures	Muscle hypertrophy	IQ & cognitive performance	Eyes	MRI and/or ultrasound	Other	Reference
1	LGMD	+	2,5y	Turkish	×28	reduced aDG expression	walks alone (at 3y), difficulty in climbing stairs, slower running than peers, stopped walking at 18y	achilles	thighs, calves	IQ 65		normal		[8]
2	LGMD	not known	1y	Turkish	N/A	reduced aDG expression	walks alone (at 3y), difficulty in climbing stairs, slower running than peers, stopped walking at 14y	achillles, spine, neck	No	IQ 50		normal	mild microcephaly	[8]
3	LGMD	+	3y	Turkish	×40	reduced aDG expression	walks alone (at 3y), difficulty in climbing stairs, slower running than peers, stopped walking at 13y	achilles	No	IQ 50		normal		[8]
4	LGMD	+	3y	Turkish	×20	reduced aDG expression	walks alone (at 3y), difficulty in climbing stairs, slower running than peers, walks with difficulty at 16y	achilles	calves	IQ 55		not studied		[8]
5	LGMD	+	2y	Turkish	×40	reduced aDG expression	walks alone (from 4y to 17y), difficulty in climbing stairs,stopped walking at 17y	achilles, spine, neck	thights,calves,trunk,arms	IQ 50		normal		[8]
6	LGMD		18 m	German	×39	dystrophic pattern, aDG normal	delayed motor development, sitting at 12 m, walking 24 m; slight proximal limb weakness	achilles	calves	delayed speech development, first words at 2,5y; learning disability, dyskalkulie	normal	normal	microcephaly, LV dysfunction (FS 26%), mild restriction in spirometry (FVC 69%)	
7	LGMD		18 m	German	×10	myopathic pattern, reduced aDG expression	Walking at 22 m; proximal limb weakness, general hypotonia; at 9 years: climbs stairs (4 floors), walks long distances, difficulties in motor coordination	No	calves	IQ 68; able to count to 20 and to read some words		normal	secondary microcephaly, cardiac diagnostic normal; muscle pain 1x per month	[14]
8a	LGMD	not known		Turkish	×22		moderate motor impairment, LGMD-like clinical picture		calves	moderate cognitive impairment		normal	crampi on physical activities, elevated GOT/GPT/LDH, microcephaly	this study
8b	LGMD			Turkish	×28	dystrophic pattern, aDG not studied	moderate motor impairment, LGMD-like clinical picture		calves	moderate cognitive impairment		not studied	crampi on physical activities, elevated GOT/GPT/LDH, microcephaly	
9	LGMD		1 m	German	×55	myopathic pattern	walks alone (from 3,5y to 29y), difficulty in climbing stairs, proximal limb weakness, Gowers sign with 4y; general hypotonia in infancy, delayed motor dev.	achilles, spine, neck, ellbows	calves, trunk, tongue	moderate cognitive impairment	normal	normal	secondary microcephaly, orofacial dysfunction	this study
10	LGMD		3 m	German	×21	reduced aDG expression	no walking/standing at 22 m			no words at 20 m		normal	microcephaly, hypersalivation	this study
11	LGMD	+	1y	Turkish	×10	no biopsy	walked at 4 y, slower than peers	No	calves	IQ 50	normal	normal	secondary microcephaly, HCF 51 cm at 7y	this study
12	LGMD	+	9 m	Turkish	×20	reduced aDG expression	walked at 3y, slow walker	No	calves	IQ 55	normal	normal	secondary microcephaly, HCF 42 cm at 1y	this study
13a	LGMD	+	1y	Turkish	×40	no biopsy	walked at 3y, stopped walking at 15y	achilles, spine, neck	calves, trunk, tongue	IQ 55	normal	normal	secondary microcephaly, HCF 49 cm at 6y	this study
13b	LGMD	+	1y	Turkish	×20	no biopsy	walked at 4 y, slower than peers	No	calves	IQ 55	normal	not studied	secondary microcephaly, HCF 48,5 cm at 12y	
14	LGMD	not known	1y	Turkish	×10	no biopsy	walked at 1y, slow walker	No	No	IQ 55	normal	normal		this study
15	LGMD	+	18 m	Turkish	×25	reduced aDG expression	walked at 5, stopped at 13 y	achilles, spine, neck	calves	IQ 50	normal	normal	secondary microcephaly, HCF 51 cm at 12y	this study
16	LGMD	+	1y	Turkish	×25	no biopsy	walked at 4 y, slower than peers	No	calves	IQ 50	normal	not studied	secondary microcephaly, HCF 47,5 cm at 4y	this study
17	LGMD	not known	1y	Turkish	×20	reduced aDG expression	walked at 6 y, slower than peers	achilles	calves	IQ 55	normal	normal	secondary microcephaly, HCF 48,5 cm at 8y	this study
18	WWS		n	Gipsy	×25		neonatal: severly hypotonic, adynamic; no psychomotor development				Bu; Gl; dense opacities of anterior parts	HC; Lis; BS; CD; CC; fused frontal lobes/ventricles/basal ganglia	cryptorchism, kidney cysts, epileptic seizures	[15]
19	WWS	+	p	Turkish	×10	dystrophic pattern, merosin normal	prenatal: HC, polyhydramnios, red. Fetal movemets: neonatal: gen. Weakness& hypotonia&hyporeflexia; markly delayed development, no sitting	No	No		Ca; RD	HC; Lis; BS; CD	tube feeding as neonate, VP shunting at 10d. Myoclonic epilepsy, drug resistant	[11]
20	WWS	+	p	Indonesian			died at 4 days of age in Indonesia				CA	HC	previous pregnancy with massive HC	this study
21a	WWS		p	German	×30		neonatal: gen. Weakness&hypotonia, reduced limb movements, could not lift up legs from underground				CA, MO; RD	HC; Lis; BS; CD; CC; occipital EC	nasogastric tube feeding as neonate; tonic and spasm-like epileptic seizures starting at 4 weeks of age	this study
21b	WWS		p									fetal ultrasound: HC, occipital EC	termination of pregnancy	
21c	WWS		p									fetal ultrasound: exencephaly	termination of pregnancy	
22a	WWS		p	German		Fetus of 18 weeks GA: premature muscle structure, absent aDG						fetal ultrasound: HC	termination of pregnancy	this study
22b	WWS		p									fetal ultrasound: HC	termination of pregnancy	
22c	WWS		p									fetal ultrasound: HC	termination of pregnancy	
23a	WWS		p	German								fetal ultrasound: HC	termination of pregnancy	this study
23b	WWS		p									fetal ultrasound: HC	termination of pregnancy	
23c	WWS		p									fetal ultrasound: HC	termination of pregnancy	
24	WWS	+	p	Turkish	×44	no biopsy	neonatal: gen. Weakness&hypotonia, reduced spontanous movements, could not lift up legs from underground	No	No		bilateral Bu, Gl, Ca; unilateral RD	HC; Lis; BS; CD; CC	unilateral clump feet; tube feeding as neonate; VP shunting with 2 months due to progressive HC; cryptorchism	this study
25	WWS	+	p	Turkish	×29		at 22 m severe muscular hypotonia, red. Spontaneous movements, no head control, no grasping	No	No	at 22 m no eye contact, no words, no reaction to external stimulation	MO; secondary Gl	fetal ultrasound: HC; reduced gyration; CD; EC; postnatal ultrasound: Lis; HC; CD; EC	microcephaly at birth; tube feeding as neonate; probable defective hearing; VP shunting with 2 months due to progressive HC; spasm-like epileptic seizures, therapy with valproic acid	this study
26	MEB-like		n	African	×26	reduced aDG expression	neonatal: muscular hypotonie with reduced limb movements; at 4 y no walking, able to turn around, to hold head when sitting, grasp things	achilles		global developmental delay (cognition, speech, motor)	cStr	HC; BS; CD	VP shunting; swallowing problems	this study
27	WWS		p	German								fetal MRI & ultrasound: HC, BS, CD	termination of pregnancy	this study

*p* prenatal, *n* neonatal, *m* months, *y* years, *aDG* α-dystroglycan, *aDG* α-dystroglycan, *Gl* glaucoma, *HCF* head circumference, *Lis* lissencephaly, *Ca* cataracts, *HC* hydrocephalus, *VP* ventriculoperitoneal, *MO* microphthalmus, *BS* brainstem involvement, *LV* left ventricular, *RD* retinal detachment, *CD* cerebellar dysplasia, *FS* fractional shortening, *Bu* Buphthalmos, *CC* hypoplastic corpus callosum, *FVC* forced vital capacity, *cStr* convergent strabismus, *EC* encephalocele

#### WWS/MEB cohort

This cohort consisted of one family with MEB-like phenotype and 9 families (15 patients) with WWS. 8/9 WWS families showed prenatal onset with ventricular dilatation in the ultrasound and/or MRI examination, one family had no medical follow-up during pregnancy. In 9 fetuses from 4 different families prenatal diagnosis of WWS resulted in premature termination of pregnancy. Six WWS patients from 6 independent families were born alive. All these neonates had general muscular weakness with reduced limb movements reported in 4/6 patients. 4/6 WWS neonates needed nasogastric tube feeding due to feeding difficulties (no information available in patient 18; patient 19 died at 4 days of age). In 4/6 WWS patients epilepsy starting in infancy was reported with myoclonic, tonic or infantile spasms-like seizures. All WWS patients showed severe ophthalmologic anomalies including congenital cataracts (reported in 3/6 families), microphthalmus (1/6), buphthalmus (2/6), and retinal detachment (2/6). All WWS patients had severe brain malformations detected by ultrasound and/or MRI: hydrocephalus internus (reported in 6/6 families), lissencephaly type II (4/6), hypoplasia of the pons and/or brainstem (3/6), cerebellar hypoplasia (5/6), hypoplasia of the corpus callosum (2/6), encephalocele (2/6). 2/6 WWS patients had ventriculoperitoneal shunting in infancy due to increasing ventricular dilatation. In 2/6 WWS patients death in infancy was reported at the age of 2.5 and 7 months, respectively.

The MEB-like patient also had muscular weakness and reduced limb movement as neonate and severe global developmental retardation in the further course. Brain imaging revealed hypoplasia of pons and vermis and an extensive hydrocephalus internus leading to ventriculoperitoneal shunting in infancy. There was a convergent strabismus in the ophthalmologic examination but no structural eye anomalies.

In 5/10 patients of the WWS/MEB cohort CK values were available and were markedly elevated with 10- to 30-fold the upper reference limit. Muscle biopsy was performed in 3/10 patients yielding a dystrophic pattern and/or reduced aDG expression in the immunofluorescent staining.

#### Case report WWS

**Fig. 1:**
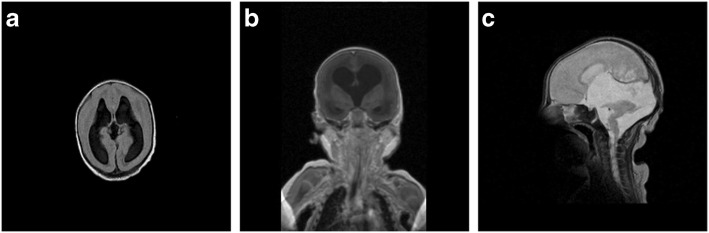
Cerebral MR-imaging of a patient with Walker-Warburg syndrome (WWS) at the age of 12 days (patient 21 a) showing bilateral enlargement of the internal ventricles and cobblestone lissencephaly (Type 2) with lack of gyration (**a**: axial fluid attenuated inversion recovery [FLAIR]; **b**: coronal magnetization-prepared rapid gradient echo [MP-RAGE]). The mid-sagittal section scan (**c**) demonstrates kinking of the brainstem and cerebellar dysplasia with absent vermis. Note the small corpus callosum (T2 weighted turbo spin echo [TSE])

Patient 21a was a German girl from non-consanguineous healthy parents with prenatal ultrasound diagnosis of a severe brain malformation with ventricular dilatation, hypoplasia of the cerebellar vermis, agenesis of the corpus callosum and occipital encephalocele. While similar brain defects in two preceding pregnancies had led to termination of pregnancy, the mother this time decided to carry the child to term. At birth at a gestational age of 39 weeks the female neonate showed severe muscular weakness with reduced spontaneous movements but without contractures. She was microcephalic with a head circumference of 31 cm. Due to weak sucking she was initially fed via a nasogastric tube. The serum CK level was 5338 U/l. Ophthalmologic examination showed bilateral microphthalmus and cataracts. MR imaging confirmed the severe brain malformation with cobblestone lissencephaly, hydrocephalus, hypoplastic corpus callosum, pontocerebellar hypoplasia and occipital encephalocele (Fig. 1). An intermittent slowing of the left hemisphere in the EEG was found. At the age of 3 months tonic and infantile spasm-like seizures developed and were successfully treated with valproic acid and sultiame. At the age of 4 months she was markedly hypotonic with a frog-like supine position, increasing paucity of spontaneous movements and lack of head control. She died unexpectedly at the age of 7 months at home, autopsy revealed pneumonia as cause of death.

#### LGMD cohort

This cohort consisted of 17 families (19 patients) with LGMD. All patients of this cohort showed symptoms of muscular dystrophy with muscular hypotonia, proximal limb weakness and delayed motor development. 16/17 LGMD families were reported with onset of symptoms at the age of 1 month to 3 years (no information on symptom onset available in family 8). In 10/17 LGMD families contractures of the Achilles tendon were reported and a rigid spine syndrome in 5/17 patients. Calves muscle hypertrophy was found in 13/17 families. All patients were cognitive impaired. IQ levels were available in 14/17 patients and ranged from 50 to 68. Microcephaly was found in 12/17 families. Cerebral MRI was performed in 15/17 families and no patient had a CNS malformation. No patient showed structural ophthalmologic anomalies. 16/17 patients had markedly elevated CK values with maximum CK values ranging from 10- to 55-fold of the upper limit (CK value not available in patient 2). For confirmation of diagnosis a muscle biopsy was performed in 13/17 families showing reduced aDG expression in 11/17 samples (not studied in 2 patients).

#### Case report LGMD

**Fig. 2:**
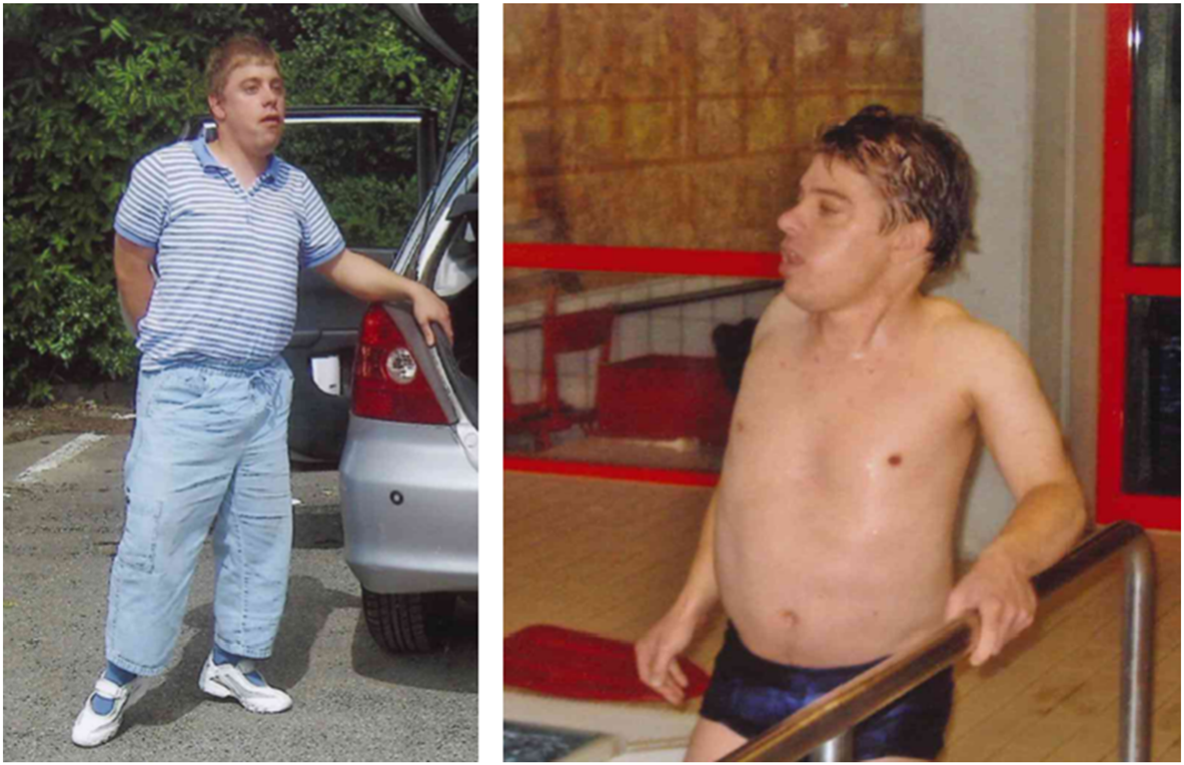
Adult patient with limb girdle muscular dystrophy with mental retardation (patient 9)

Patient 9 was the third child of non-consanguineous German parents with no relevant family history besides the 3 unexplained miscarriages the patient’s mother had before. During pregnancy the mother assumed slightly reduced fetal movements in an otherwise uneventful pregnancy. There was a normal delivery at a gestational age of 37 weeks, birth weight was 2900 g (90th percentile) and head circumference 33 cm (25th percentile). Around 4 weeks of age the mother first noted muscular hypotonia. The motor milestones were markedly delayed with acquisition of unsupported sitting at 16 months and walking at 3.5 years. From the age of 4 years on a proximal limb weakness became evident with a positive Gowers’ sign. In the following years the patient’s motor abilities stabilized with independent ambulation; he was able to ascend stairs slowly with holding the handle. In his late 20ies the motor functions started to deteriorate and he became wheel chair dependent around 30 years of age. Pseudohypertrophy of the calves was at first documented at the age of 4 years and subsequently occurred also in his thighs, trunk and arms. He had an increased lumbar lordosis and severe contractures of the ankles, spine and neck as well as mildly of the elbows (Fig. 2). Surgery of bilateral ankle contractures was performed at the age of 12 years and improved walking. His intellectual development was severely disturbed from early childhood on. At 4 years of age only a few words could be pronounced clearly, the patient never learned writing or reading and independent activities never could be performed. Secondary microcephaly developed in the first 4 years of life, but epileptic seizures did never occur. Cerebral MR imaging was reported to be normal. While orofacial weakness and hypersalivation were treated with speech and language therapy from early childhood on, there was no prominent facial weakness and a normal ophthalmologic status. At the age of 30 years the left ventricular function was normal. Repeatedly, the CK values were markedly elevated (1644–9860 U/l). A first muscle biopsy and electromyography performed at the age of 4 years revealed a myopathic pattern and led to the initial suspicion of Duchenne muscular dystrophy. In a second muscle biopsy at 11 years of age the expression of dystrophin was normal as was the genetic analysis of the *dystrophin* gene. Expression of glycosylated α-dystroglycan was not studied in any of the muscle biopsies. The patient was seen in different pediatric and adult neuromuscular centers on a regular basis. Finally, at the age of 32 years the patient’s family again searched for a genetic diagnosis at the Munich neuromuscular center and clinical diagnosis of *POMT1*-related LGMD could be genetically confirmed by identification of compound heterozygous *POMT1* mutations.

### Genetic findings

**Fig. 3:**
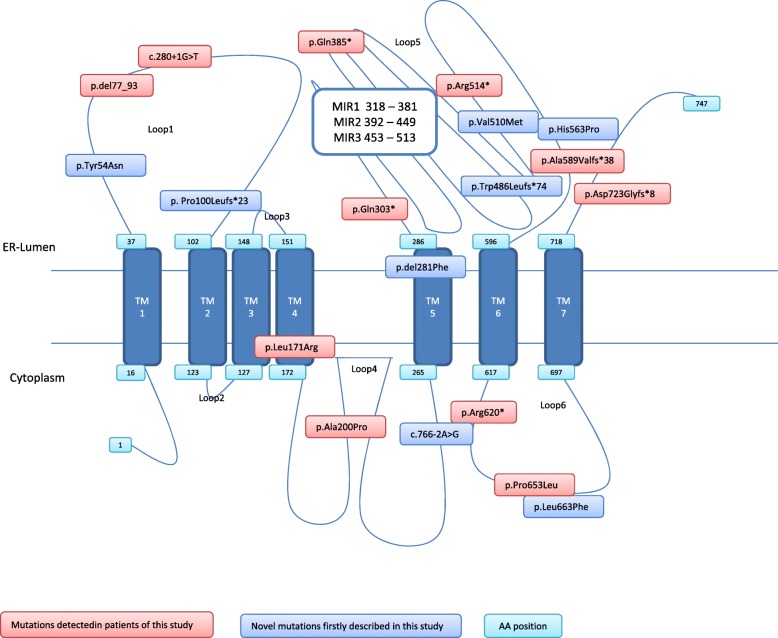
*POMT1* mutations detected in patients of this study. Mutations can be located in the cytoplasma, in transmembranous domains, or in the endoplasmatic reticulum (ER). MIR: motif in overlapping homologous superfamilies (IPR016093)

In all patients included in our study two *POMT1* mutations were recognized with 9 families carrying compound heterozygous mutations and 18 families with homozygous mutations. We identified 10 previously reported and 8 novel *POMT1* mutations (Table 2; Fig. 3). A list of all causative *POMT1* mutations reported to date is provided as Additional file 1.

**Table 2:** Summary of *POMT1* mutations identified in patients of this study

Patient	Diagnosis	Exon/Intron	Nucleotide change	Predicted amino acid change	Mutation type	Reference
1	LGMD	Exon 7	c.598G > C	Ala200Pro	Missense	[8]
		Exon 7	c.598G > C	Ala200Pro	Missense	[8]
2	LGMD	Exon 7	c.598G > C	Ala200Pro	Missense	[8]
		Exon 7	c.598G > C	Ala200Pro	Missense	[8]
3	LGMD	Exon 7	c.598G > C	Ala200Pro	Missense	[8]
		Exon 7	c.598G > C	Ala200Pro	Missense	[8]
4	LGMD	Exon 7	c.598G > C	Ala200Pro	Missense	[8]
		Exon 7	c.598G > C	Ala200Pro	Missense	[8]
5	LGMD	Exon 7	c.598G > C	Ala200Pro	Missense	[8]
		Exon 7	c.598G > C	Ala200Pro	Missense	[8]
6	LGMD	Exon 15	c.1456dupT	Trp486Leufs*74	Frame shift	this study
		Exon 3	c.160 T > A	Tyr54Asn	Missense	this study
7	LGMD	Exon 18,19	partial deletion Exon 18–19	A589Vfs*38	Frame shift	[14]
		Exon 6	c.512 T > G	Leu171Arg	Missense	[14]
8 a, b	LGMD	Exon7	c.598G > C	Ala200Pro	Missense	[8]
		Exon7	c.598G > C	Ala200Pro	Missense	[8]
9	LGMD	Exon 19	c.1987C > T	Leu663Phe	Missense	this study
		Exon 20	c.2167dupG	Asp723Glyfs*8	Frame shift	[4, 7, 10]
10	LGMD	Exon 19	c.1958C > T	Pro653Leu	Missense	[10]
		Exon 15	c.1456dupT	Trp486Leufs*74	Frame shift	this study
11	LGMD	Exon 7	c.598G > C	Ala200Pro	Missense	[8]
		Exon 7	c.598G > C	Ala200Pro	Missense	[8]
12	LGMD	Exon 7	c.598G > C	Ala200Pro	Missense	[8]
		Exon 7	c.598G > C	Ala200Pro	Missense	[8]
13a, b	LGMD	Exon 7	c.598G > C	Ala200Pro	Missense	[8]
		Exon 7	c.598G > C	Ala200Pro	Missense	[8]
14	LGMD	Exon 7	c.598G > C	Ala200Pro	Missense	[8]
		Exon 7	c.598G > C	Ala200Pro	Missense	[8]
15	LGMD	Exon 7	c.598G > C	Ala200Pro	Missense	[8]
		Exon 7	c.598G > C	Ala200Pro	Missense	[8]
16	LGMD	Exon 7	c.598G > C	Ala200Pro	Missense	[8]
		Exon 7	c.598G > C	Ala200Pro	Missense	[8]
17	LGMD	Exon 7	c.598G > C	Ala200Pro	Missense	[8]
		Exon 7	c.598G > C	Ala200Pro	Missense	[8]
18	WWS	Intron 4	c.280 + 1G > T	p.del77_93 (delExon4)	Donor splice site	[15, 31]
		Intron 4	c.280 + 1G > T	p.del77_93 (delExon4)	Donor splice site	[15, 31]
19	WWS	Exon 15	c.1540C > T	Arg514*	Nonsense	[11]
		Exon 15	c.1540C > T	Arg514*	Nonsense	[11]
20	WWS	Exon 18	c.1558C > T	Arg620*	Nonsense	[33]
		Exon 18	c.1558C > T	Arg620*	Nonsense	[33]
21 a, b, c	WWS	Exon 20	c.2167dupG	p.Asp723Glyfs*8	Frame shift	[4, 7, 10]
		Exon 12	c.1153C > T	Gln385*	Nonsense	[4]
22 a, b, c	WWS	Exon 9	c.842_844delTCT	del281Phe	In frame	this study
		Exon 20	c.2167dupG	p.Asp723Glyfs*8	Frame shift	[4, 7, 10]
23 a, b, c	WWS	Exon 11	c.1153C > T	Gln385*	Nonsense	[4]
		Exon 20	c.2167dupG	p.Asp723Glyfs*8	Frame shift	[4, 7, 10]
24	WWS	Exon 20	c.2167dupG	p.Asp723Glyfs*8	Frame shift	[4, 7, 10]
		Exon 20	c.2167dupG	p.Asp723Glyfs*8	Frame shift	[4, 7, 10]
25	WWS	Exon 9	c.907C > T	p.Gln303*	Nonsense	[4]
		Exon 9	c.907C > T	p.Gln303*	Nonsense	[4]
26	MEB-like	Exon 15	c.1528G > A	p.Val510Met	Missense	this study
		Exon 17	c.1688A > C	p.His563Pro	Missense	this study
27	WWS	Exon 5	c.299delC	p.Pro100Leufs*23	Frame shift	this study
		Intron 8	c.766-2A > G	p.?	Acceptor splice site	this study

#### WWS/MEB cohort

5/9 WWS families were found to have homozygous *POMT1* mutations (3 Turkish, 1 Indonesian, 1 Gipsy family); the other families were compound heterozygous. 9/9 WWS families had 2 *POMT1* mutations considered to severely disrupt transcript or protein synthesis: 1/9 patient (family 18) had a homozygous donor splice site mutation expected to alter the splicing of intron 3 and/or neighboring exons [15]. In 4/9 families (families 19, 20, 24, 25) homozygous nonsense mutations were identified predicted to result in premature protein termination. In 2/9 families (families 21, 23) 2 compound heterozygous nonsense mutations were found each leading to a premature stop codon. 1/9 patient (family 27) was compound heterozygous for a splice site mutation and a frame shift mutation leading to a premature stop codon. 1/9 family (family 22) was compound heterozygous for a nonsense mutation with presumed premature protein termination and an in-frame mutation presumed to result in deletion of a phenylalanin residue at position 281.

The MEB patient with African parents had compound heterozygous missense mutations. One mutation (p.His563Pro) was of maternal origin and was not described before. Unfortunately, no material of the father could be obtained.

#### LGMD cohort

All LGMD patients had at least 1 missense mutation. 13/17 families were homozygous for the p.Ala200Pro mutation described before as an ancestral founder mutation in Turkish families with a distinct phenotype [8]. 4/17 patients were compound heterozygous for a missense mutation and a frame-shift mutation predicted to result in premature protein termination.

## Discussion

### Phenotypic variability of disorders associated with *POMT1* mutations: the most severe end of the spectrum

All patients in our WWS/MEB cohort displayed a most severe phenotype with striking muscular weakness/hypotonia with prenatal or neonatal onset as described in literature before [1, 4]. Available serum creatine kinase values were markedly elevated in all WWS/MEB patients (1850–5338 U/l). As stated 1989 in Dobyns’ WWS criteria all neonates of our families presented with severe anterior and/or posterior eye abnormalities, most frequently congenital cataracts, buphthalmus and retinal detachment [5, 17]. Surprisingly and in contrast to former studies [5], arthrogryposis or joint contractions were not notified in any WWS neonate besides bilateral clump feet in one patient. Epilepsy seemed a frequent finding with different seizure types occurring (myoclonic, tonic, spasm-like) and potentially drug resistance. More detailed information on the antiepileptic drug management was available for one patient only who achieved seizure control on valproate and sultiame. As reported before, life expectancy was severely shortened with death occurring before 1 year of age in 2 patients [6].

**Fig. 4:**
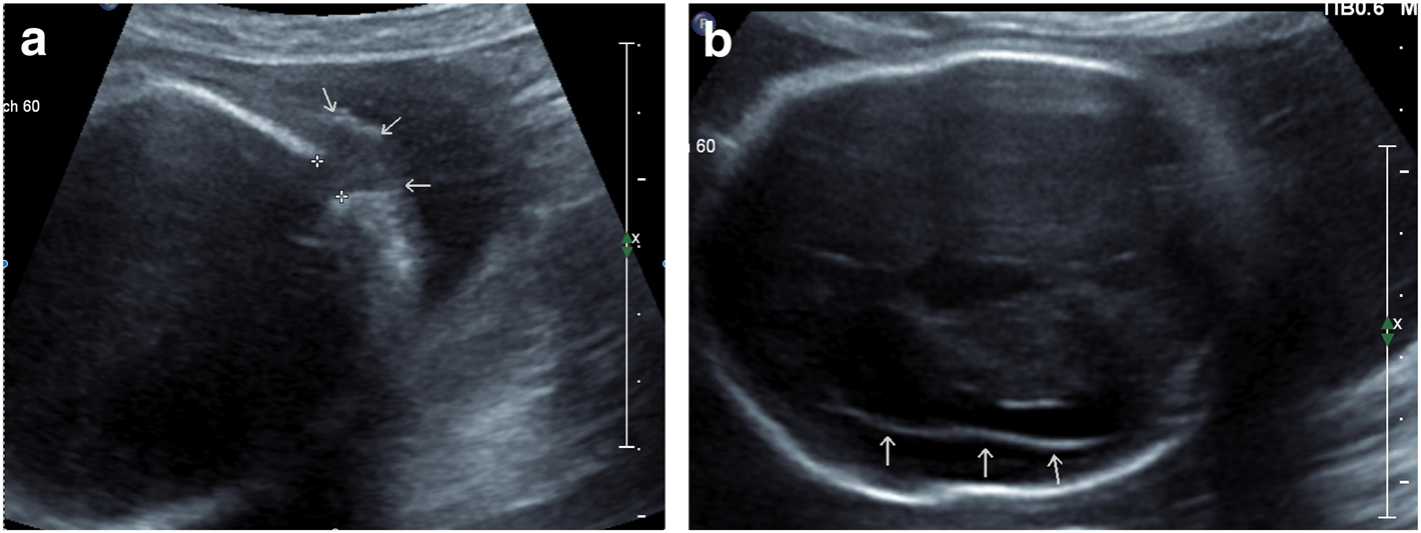
Prenatal ultrasound at 31 weeks of gestational age of a patient with Walker-Warburg syndrome (patient 25). **a** shows an occipital encephalocele (arrows) and **b** reveals absent gyration due to lissencephaly (arrows)

Constant structural brain malformations in our WWS/MEB cohort included severe ventricular dilatation, cobblestone lissencephaly and hypoplasia of brainstem and cerebellum. Very similar brain involvement was found in WWS patients with genetically confirmed dystroglycanopathy due to mutations in other genes like *POMT2*, *LARGE*, *POMGnT1* and *FUKUTIN,* respectively. A gene specific pattern of brain malformations in patients with dystroglycanopathies appears not to exist [1, 18]. This assumption is supported by a French pathological study of aborted fetuses with cobblestone lissencephaly that found *POMT1* being the most frequent causative gene and also described an overlapping phenotype independent of the affected gene [19, 20]. Remarkably, neural tube defects ranging from meningocele to major occipital meningoencephalocele could be linked to *POMT1* in 6/7 cases with an identified mutation [19]. In accordance, in our WWS cohort occipital encephalocele was detected in 2 families (Fig. 4).

**Fig. 5:**
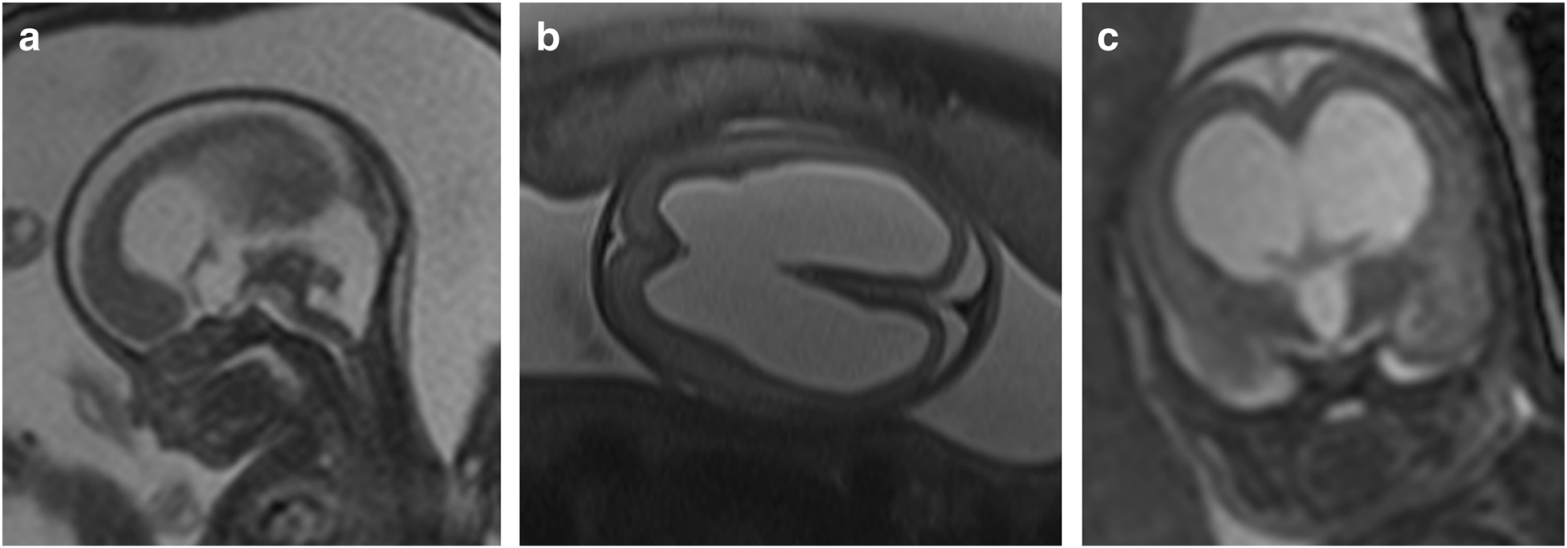
Prenatal MR-imaging at 23 weeks of gestational age of a patient with Walker-Warburg syndrome (patient 27). **a** shows kinking of the brainstem and flattening of the pons. **b** (axial section) and **c** (coronal section) demonstrate extensive enlargement of the internal ventricles (**a**, **c**: true fast imaging with steady state precession [TrueFISP]; **b**: half-fourier acquisition single-shot turbo spin echo [HASTE]). **Of note**: mostly absent gyration is regular at that early week of gestation, thus diagnosis of lissencephaly here not appropriate

In our WWS/MEB cohort 4 families had a prenatal diagnosis of WWS resulting in termination of pregnancy (Fig. 5). In 3 of those families fetal WWS with rather similar prenatal sonographic presentation was diagnosed even in 3 consecutive pregnancies. One family with termination of the first 2 pregnancies due to confirmed *POMT1*-related WWS decided to carry out the baby in the third pregnancy despite once more sonographically suspected WWS. This neonate presented with a typical clinical course of a severe WWS as described in the result section. These data further support an identical intrafamiliar course of disease in families with more than one affected child, which was noted before in other families [6].

### Phenotypic variability: milder forms

In accordance with earlier reports [8, 10, 21, 22] all patients of our LGMD cohort had a typical LGMD phenotype with axial and proximal limb weakness, difficulties in motor coordination and performance and markedly elevated serum creatine kinase values. Muscle biopsy was performed in most LGMD patients (13/17) allowing disease categorization as dystroglycanopathy. None of the LGMD patients had structural brain malformation on the cerebral MRI, but moderate to severe cognitive impairment was present in all patients. This feature represent a key symptom discriminating those patients from most other LGMD forms [23]. Additionally, microcephaly was a very common finding and could serve as another diagnostic clue whereas neither epilepsy nor ophthalmologic problems were reported in any of the LGMD patients from our cohort. Likewise*,* the few individuals described with *POMT2*–related LGMD (LGMD2N) also had cognitive impairment [1, 24, 25]. This might be explained by the close functional relation of the two encoded proteins (protein O-mannosyltransferase 1 and 2) which form an enzyme complex catalyzing the first step of glycosylation of aDG [26, 27].

The largest LGMD subgroup in our cohort consisted of as many as 15 patients who were homozygous for the *POMT1* founder mutation p.Ala200Pro ultimately defining LGMD2K [8]. These patients presented specifically with delayed motor development with independent walking at 1 to 6 years, calves muscle pseudohypertrophy and shortened Achilles tendon in many patients and rigid spine syndrome in some. In 2 families with 2 affected patients both siblings had an identical phenotype. In addition to the patients’ characteristics described 2005 by Balci et.al. follow-up data over 13 years provided by one of the authors (HT) now revealed progression of muscular weakness in all patients leading to the loss of walking ability between the age of 13 to 18 years [8]. One patient (patient 9) of our cohort even remained ambulatory to the age of 30 years. Similarily, two sisters of similar age with adult onset LGMD sustained a marked decline in muscle strength and function around the age of 30 years [22]. This emphasizes the progressive nature of the disease also in milder LGMD phenotypes, ultimately resulting in loss of ambulation at a variable age.

### *POMT1* genotypes and genotype-phenotype correlation

The presumed underlying biochemical mechanism for a more and less severe phenotype and for the presence of a genotype-phenotype correlation is the reduction in enzyme activity of the protein O-mannosyltransferase-1 (POMT1) at a variable degree. The residual enzyme activity of the mutant gene products is inversely correlated with the severity of the phenotype [14, 28]. It is assumed to critically depend on the type and location of the *POMT1* mutation as highlighted in several previously reported patients [1, 7, 8, 10, 28–31]. In general, biallelic *POMT1* loss of function mutations due to nonsense mutations or frameshift mutations with premature protein truncation result in a severe WWS phenotype while the presence of at least one missense mutation appears to be sufficient to result in milder phenotypes.

In the WWS subgroup of our study all but one family were found to be homozygous or compound heterozygous for different nonsense mutations each leading to premature protein truncation. In one family (family 22) with a severe WWS phenotype and three affected fetuses there was the special situation of compound heterozygosity for the well-established nonsense mutation p.Asp723Glyfs*8 [10] and an in-frame mutation (c.842_844delTCT) predicted to result in the deletion of a phenylalanine residue at position 281 (p.del281Phe). Phenylalanine at that position is highly conserved across multiple species down to baker’s yeast with an allele frequency of 0,0053% in the ExAC (1.0) browser. It is located in the transmembrane domain of the protein and thus at a location reported to be related to more severe phenotypes [10]. Moreover, deletion of a phenylalanine residue at another position (p.del60Phe) was described before in a fetus with WWS [19]. Therefore we hypothesize that the special location of this in-frame mutation (Fig. 3) and the specific function of this phenylalanine residue causes the WWS phenotype in this family.

The impact of the mutation type and location within the different domains of the POMT1 protein can be specifically studied in the LGMD cohort (Fig. 3). The homozygous founder mutation p.Ala200Pro identified in the largest subgroup of 15 Turkish patients with LGMD is located in the cytoplasm (loop 4) [8]. The missense mutation p.Pro653Leu in the cytoplasmatic loop 6 was reported before to reduce the phenotypic severity in compound heterozygous patients [10]. We found another 3 families with a milder phenotype due to compound heterozygosity for a frameshift mutation and a missense mutation: the mutation c.1987C > T (p.Leu663Phe) is situated in the cytoplasmatic loop 6 very close to the above mentioned p.Pro653Leu substitution. The mutation c.512 T > G (p.Leu171Ala) is positioned at the connection of a cytoplasmatic loop to a transmembrane domain. In contrast, the mutation c.160 T > A (p.Tyr54Asn) is positioned in loop 1 within the lumen of the endoplasmatic reticulum (ER). Tyrosine at position 54 is highly conserved across species up to Baker’s yeast. Physicochemically there is a great difference between tyrosine and the substitute asparagine. However, mutations in loop 1 have been linked before to both WWS phenotypes and milder forms of LGMD, respectively [7, 10, 22]. This again emphasizes the importance of the mutation location for the resulting phenotype.

Of particular interest is the genotype of the only MEB patient of our cohort. This girl was compound heterozygous for 2 missense mutations located in the ER in a protein domain considered essential for the catalytic enzyme activity. Both substituted amino acids are highly conserved across species down to fruitfly (p.His563Pro) and to baker’s yeast (p.Val510Met), respectively. In silico analysis, allele frequency and physicochemical differences for each of the substituted amino acid are contradictory [see Additional file 2] and both mutations have not been described before in affected patients. We hypothesize that the location in the catalytic active domain critically affects the enzyme function resulting in a more severe phenotype compared to other individuals carrying 2 missense mutations located at other protein domains.

Remarkably, the mutation c.2167dupG was found in 4/8 unrelated families with Caucasian (German) origin suggesting a mutational hotspot.

### Limitations of the study

Due to the rareness of dystroglycanopathies the patients included in this study were referred to us for genetic analysis from different pediatric, genetic and neurologic centers and therefore clinical examinations were performed by different physicians involved in the patient’s medical care; however, the referring centers were particularly experienced in treating patients with neuromuscular diseases. A muscle biopsy demonstrating hypoglycosylation of α-dystroglycan formerly constituting the diagnostic gold standard was performed in 16 of 27 families only. However, those patients without muscle biopsy had a highly suggestive clinical phenotype for a dystroglycanopathy. As there was no application of next generation sequencing in most patients and identification of two *POMT1* mutations was defined an inclusion criterion, affected individuals with only one identified mutation have not been included in this study, because their heterozygous mutation may be present by chance. Furthermore, heterozygous *POMT1* exon deletions may have been missed prior to the introduction of NGS due to methodical limitations in the genetic analysis.

## Conclusions

Patients with *POMT1*-related disorders present with a variable phenotype and a broad range of severity. They all have markedly elevated serum creatine kinase values and show a progressive course of disease. Milder LGMD phenotypes as well have a decline in muscle strength and function finally leading to the loss of walking ability at a variable age. In LGMD patients functional brain involvement with cognitive impairment and microcephaly occurs constantly and can be helpful in the differential diagnosis. In contrast, structural brain and eye involvement is a key feature in WWS/MEB patients. Intrafamiliar rather identical clinical courses can be expected in both LGMD and WWS phenotypes. Generally, a genotype-phenotype correlation of *POMT1*-related disorders exists. A severe WWS phenotype develops in patients carrying two mutations leading to premature protein termination. The presence of at least one missense mutation might result in a milder phenotype. However, the impact of a missense mutation on the resulting phenotype critically depends on the mutation’s type and location and thus each *POMT1* mutation should be analyzed in detail accordingly. The expanded knowledge on genotype-phenotype correlation from our study (Table 2) and the provided list of all causative *POMT1* mutation recognized by now [Additional file 1] add detailed information for a profound genetic counseling for affected families. Moreover, possible diagnostic clues for *POMT1*-related disorders are depicted in Table 3.

**Table 3:** Possible diagnostic clues in patients with *POMT1*-related disorders

A good clinical characterization remains a mandatory prerequisite greatly improving the diagnostic strategy and ultimately shortening the diagnostic yield and time to report.	
WWS should be prenatally considered in the presence of fetal ultrasound abnormalities with ventricular dilatation in combination with infratentorial and/or ocular anomalies[32].	
Diagnosis of an occipital encephalocele might be a diagnostic clue for *POMT1* as causative gene within the different genes associated with WWS.	
In the European population *POMT1* is the gene most commonly associated with WWS ([18], personal data).	
In patients with a complex brain malformation not suggestive for a specific monogenic disorder early CK analysis as a simple laboratory test could potentially guide the diagnosis of a dystroglycanopathy.	
In patients with unexplained cognitive impairment, microcephaly and muscular weakness early CK analysis may also help to identify milder clinical manifestations of dystroglycanopathy.	
A dystroglycanopathy should also be considered in any CMD or LGMD patients, negative for Duchenne or Becker muscular dystrophy, with cognitive impairment with or without structural brain malformations, prompting genetic analysis of a NGS panel including *POMT1* prior to muscle biopsy.	

## Materials and methods

### Patients

Between 2002 and 2018 blood samples of 283 patients with suspected dystroglycanopathy were referred to our center of human genetics for genetic analysis of genes linked with dystroglycanopathies. Overall, in 65/283 patients (23%) the suspected diagnosis of a dystroglycanopathy was genetically confirmed by identification of 2 mutations in a gene linked with dystroglycanopathy, most frequently in *POMT1*. Blood samples and clinical data were referred from pediatric, neurologic and genetic centers from worldwide but mostly from Germany and Turkey. In 6/27 families genetic counseling was asked for after prenatal ultrasound revealed intracranial ventricular dilatation. Clinical data was collected and patients were divided in phenotypic categories. Only patients with a characteristic phenotype and identification of 2 causative *POMT1* mutations in the genetic analysis were included in this study. Clinical evaluation of these patients included neurological and ophthalmologic examination, assessment of psychomotor development, measurement of CK values, electroencephalogram (EEG), cerebral ultrasound and brain magnetic resonance imaging (MRI). A muscle biopsy with immunohistochemical staining of α-dystroglycan had been performed in 16 patients. Clinical and genetic data of families 11–17 were obtained from HT and BBH at Hacettepe University, Ankara, Turkey. The study was approved by the local Ethics Committee of the University Regensburg (#13–101-0236). Written inform consent was obtained from all participants.

### Molecular genetics

#### Mutation analysis

Genomic DNA was prepared from peripheral blood. The entire *POMT1* coding sequence and flanking splice sites (reference sequence NM_007171.3) were amplified by PCR and analyzed for potential sequence variations by direct sequencing of PCR products. All Sanger sequencing was carried out on an ABI sequencer (Applied Biosystems, Foster City, CA, USA) for targeted conventional single gene Sanger sequencing according to the manufacture’s recommendations.

Since 2013 massive parallel sequencing methods were applied in our laboratory and positive results were confirmed by Sanger sequencing. For massive parallel sequencing genomic DNA of each patient was processed according to the Nextera Enrichment protocol (Illumina, Inc., San Diego, CA, USA). Library quantification was carried out with the High Sensitivity DNA Kit on a Bioanalyzer (Agilent Technologies, Böblingen, Germany) and the Qubit™dsDNA HS Assay Kit (Life Technologies, Darmstadt, Germany). The Library was sequenced as a 150 bp paired-end run on a MiSeq™ system (Illumina, Inc., San Diego, CA). Variant detection was performed with Illumina VariantStudio (Illumina, Inc., San Diego, CA, USA).

## Additional files

Additional file 1: List of all mutations in *POMT1.* This file lists all causative mutations identified to date in the *POMT1* gene. (XLSX 27 kb)

Additional file 2: In silico analysis of novel *POMT1* mutations. This file shows the result of the in silico analysis of pathogenicity for all novel *POMT1* mutations identified in this study. (XLSX 11 kb)

## Data Availability

All data generated or analysed during this study are included in this published article and its supplementary files.

## Abbreviations

aDG: α-dystroglycan
CK: Serum creatine kinase
DNA: Deoxyribonucleic acid
EEG: Electroencephalogram
ER: Endoplasmatic reticulum
LGMD: Limb girdle muscular dystrophy
MEB: Muscle-eye-brain disease
MR: Mental retardation
MRI: Magnetic resonance imaging
PCR: Polymerase chain reaction
POMT1: Protein O-mannosyltransferase 1, encoded by the *POMT1* gene
WES: Whole exome sequencing
WWS: Walker-Warburg syndrome
